# Quantiprot - a Python package for quantitative analysis of protein sequences

**DOI:** 10.1186/s12859-017-1751-4

**Published:** 2017-07-17

**Authors:** Bogumił M. Konopka, Marta Marciniak, Witold Dyrka

**Affiliations:** Katedra InŻynierii Biomedycznej, Wydział Podstawowych Problemów Techniki, Politechnika Wrocławska, WybrzeŻe Wyspiańskiego 27, Wroclaw, 50-370 Poland

**Keywords:** Protein sequence analysis, Python package, Quantitative properties, Quantitative recurrence analysis, n-grams

## Abstract

**Background:**

The field of protein sequence analysis is dominated by tools rooted in substitution matrices and alignments. A complementary approach is provided by methods of quantitative characterization. A major advantage of the approach is that quantitative properties defines a multidimensional solution space, where sequences can be related to each other and differences can be meaningfully interpreted.

**Results:**

Quantiprot is a software package in Python, which provides a simple and consistent interface to multiple methods for quantitative characterization of protein sequences. The package can be used to calculate dozens of characteristics directly from sequences or using physico-chemical properties of amino acids. Besides basic measures, Quantiprot performs quantitative analysis of recurrence and determinism in the sequence, calculates distribution of n-grams and computes the Zipf’s law coefficient.

**Conclusions:**

We propose three main fields of application of the Quantiprot package. First, quantitative characteristics can be used in alignment-free similarity searches, and in clustering of large and/or divergent sequence sets. Second, a feature space defined by quantitative properties can be used in comparative studies of protein families and organisms. Third, the feature space can be used for evaluating generative models, where large number of sequences generated by the model can be compared to actually observed sequences.

## Background

This is a trivial observation that functional and structural characteristics of protein sequences emerge from physico-chemical properties of amino acids. Many properties can be quantified: the well-known AAindex database [1] holds over half thousand indices. An established example of use of quantitative properties of amino acids to characterize proteins is recognition of disordered proteins, which can be well separated from ordered proteins in the feature space defined by the net absolute charge and the mean hydrophobicity [2]. A practical implementation of the method, the FoldIndex tool detects disordered regions within proteins [3]. A more complex approach consists on combining multiple quantitative properties into multi-dimensional sequence descriptors, as implemented in a Python package *propy* [4]. Quantitative properties of amino acids can also be used to generate reduced alphabets for generative and discriminative models of proteins [5, 6].

A level up in characterizing protein sequences is analysis of amino acid tuples or n-grams. For example, it was demonstrated that distribution of n-grams varied for different secondary structures [7, 8]. A newer study reported that the most available 5-grams in proteins were twice enriched in known functionally important sequence motifs [9]. Interestingly, distribution of amino acid tuples can often be approximated with the power-law distribution (the Zipf’s law) [9]. Most recently, n-gram-based random forests were sucessfully applied for accurate discrimination between amyloidogenic and non-amyloidogenic peptides [6]. Several tools for analysis of n-grams in proteins were made available, e.g. in the R language package *biogram* [10] and in the SCS Package web server [11].

A prominent feature of protein sequences are recurring patterns [12, 13], which can be quantified with the Recurrence Quantification Analysis (RQA) [14]. Early works showed that a feature space defined by the RQA parameters allowed for discrimination between functionally different protein family members or mutants [15, 16]. The technique was also used to investigate the role of hydrophobicity patterns in protein folding, aggregation and interactions [17, 18]. More recently, a Support Vector Machine on RQA parameters calculated for multiple physico-chemical properties of protein sequences was proposed for the remote homologyz detection [19].

Our contribution, the Quantiprot package, gathers multiple methods for quantitative analysis of protein sequences and makes them easily accessible to the community of computational biologists.

## Implementation

The Quantiprot package was developed using Python 2.7 [20]. The number of dependencies is kept low to make the package light-weight and easily portable to various environments. Majority of functionalities were written in pure Python, while several others require only the *numpy* package [21]. In addition, *matplotlib* [22] is required for plotting figures, *powerlaw* [23] for the power-law fitting to the n-gram distribution, *scipy.stats* [24] for calculating the Fisher exact test. The *requests* package [25] is needed only if the AAindex database is to be accessed online.

The package is built around five utility classes. The *Sequence* and *SequenceSet* classes store and manipulate sequences of various types, e.g. raw amino acid symbols and their quantitative projections. The *Feature* and *FeatureSet* classes store and perform sequence quantification actions and their chains. The *Mapping* class stores amino acid projections, reduces alphabet and performs sequence data conversions, e.g. using indices from the AAindex database.

The main utility classes are complemented by a large set of predefined quantitative metrics. In addition, user-defined metrics can be easily utilized. Finally, the package implements advanced analyses.

### Functionalities

#### Sequence manipulation

The package reads sequences in the FASTA format and stores them in the *SequenceSet* class objects. There are provided convenience functions for merging sequence sets and extracting matrices of specified columns. Moreover, there are functions for extracting subsets and compacting multiple single-value features (e.g. net charge, average hydropathy and entropy).

#### Sequence conversion

Raw amino acid sequences can be easily converted to quantitative properties (e.g. charge, hydrophobicity, propensity towards a secondary structure etc.). The user may choose a predefined mapping or any of the AAindex scales, or use own mapping. The mapping can be simplified through discretization of the quantitative property based on the user-defined or linear thresholds, or using the k-means clustering.

#### Sequence quantification

It is possible to quantify raw and converted protein sequences with a single value or to calculate sequence profiles using a sliding window. Currently implemented features range from basic measures such as property average and sum, through more sophisticated ones such as entropy, to recurrence and determinism used in the RQA. Of note, the package introduces a new RQA parameter termed *palindromism*, which is defined as the percentage of recurrence points forming antidiagonal lines in the recurrence plot.

#### Feature chaining

Conversion mappings and quantification measures are wrapped in the *Feature* class objects, which provides an easy interface for chaining. A typical complex feature may consist of a conversion from amino acid sequence to sequence of numeric values, followed by a quantification. Importantly, the *Feature* object can wrap any function that accepts the list-like inputs making it trivial to add new functionalities.

#### Patterns and n-grams counting

The package can find matches and count occurrences of arbitrary patterns (without gaps). Importantly, it is possible to define a similarity radius in several metrics in order to find inexact matches. In addition, the *analysis.ngram* module supports counting n-grams in the entire sequence set and fitting their distribution with the power-law distribution (Zipf’s law).

#### Feature space exploration

Quantiprot allows comparing two sequence sets in a 2-d feature space defined by the quantitative properties of sequences. The implemented analysis calculates a local ratio of number of sequences from each set in part of the feature space and compare it to the global ratio in the whole feature space using the Fisher’s exact test.

## Results and discussion

### Sample application

**Fig. 1:**
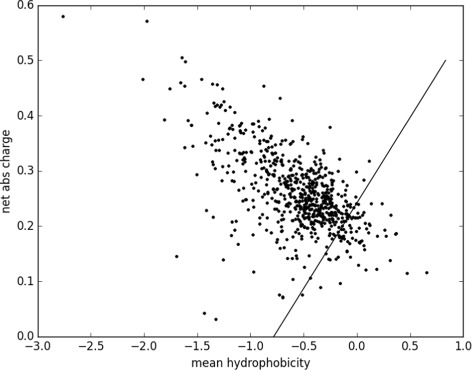
The Uversky plot for the DisProt database. The expected division line between ordered and disordered proteins is drawn



Handling of the Quantiprot package can be illustrated by generating the Uversky plot (Listing 1). The script creates a feature set consisting of the net absolute charge and mean hydropathy. Then the feature set is used to process sequences from the DisProt database [26]. Finally, feature values for all sequences are extracted and plotted using *matplotlib* (Fig. 1). Import statements in Listing 1 are omitted for the sake of brevity.

### Feature space exploration

A more advanced use of the package is to compare two protein sequence sets in a two-dimensional space defined by quantitative features of sequences. The analysis calculates a local ratio of number of sequences from each set in part of the feature space and compare it to the global ratio in the whole feature space using the Fisher’s exact test. This can be useful for comparing two populations, or two samples of a population, and also to verify if a sample generated by a model fits real observations. In practical terms, the two-dimensional feature space is divided into a square grid of cells. Then a sliding window is moved over the grid and the Fisher’s exact tests are performed in the window against the null hypothesis that the sequence distribution in the particular window is the same as in the whole feature space.

In this sample case (Listing 2), populations of amyloidogenic and non-amyloidogenic peptides in the AmyLoad database [27] are compared in the feature space defined by hydropathy and volume of amino acids. Import statements in Listing 2 are omitted.



**Fig. 2:**
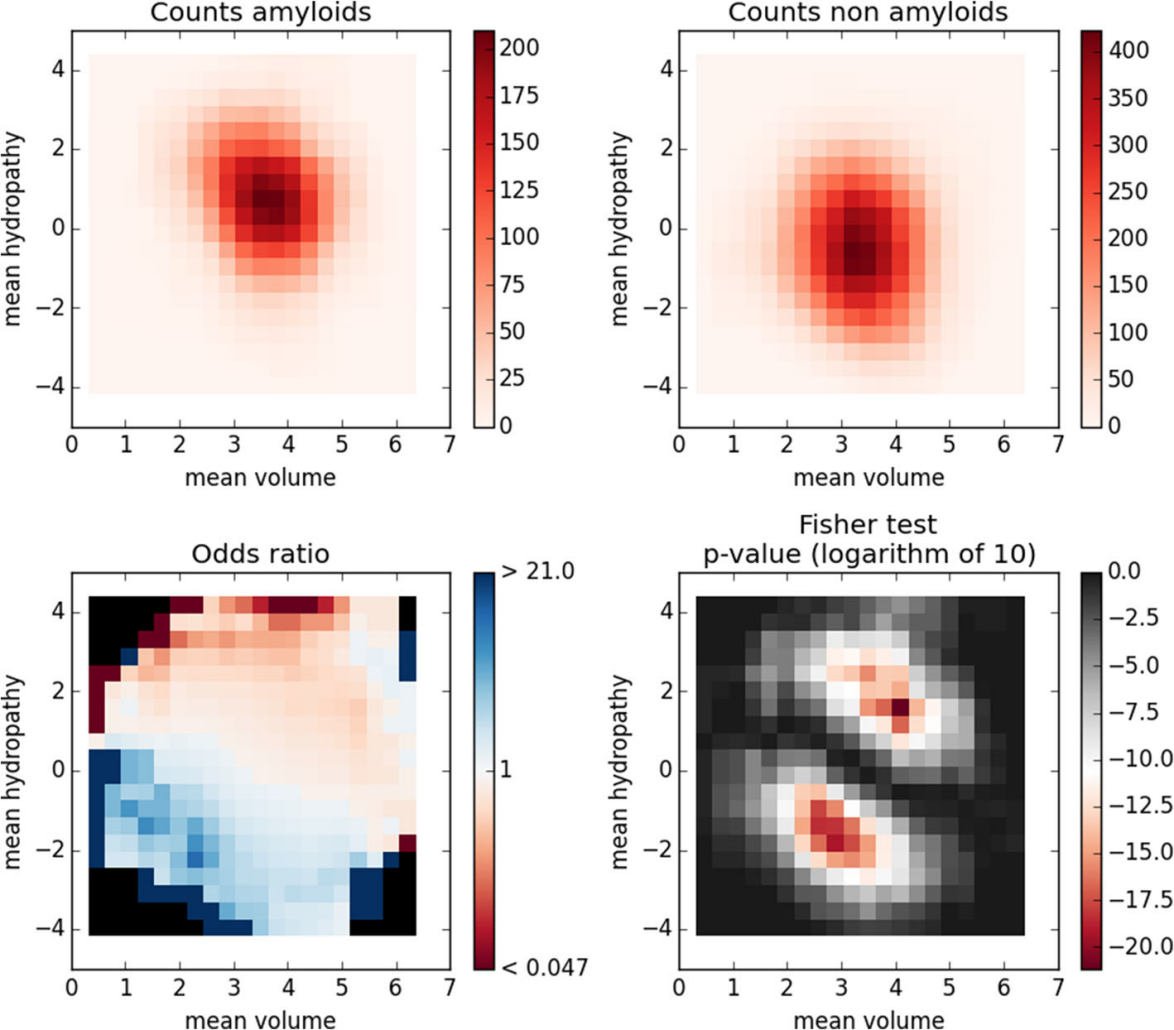
Feature space visualization for the AmyLoad database. Amyloidogenic and non-amyloidogenic peptides in the feature space defined by their mean hydrophopathy and amino acid volume. The feature space of dimensions *n*×*m* was divided into windows of dimensions $\frac {n}{k_{1}} \times \frac {m}{k_{2}}$, *k*_1_=*k*_2_=5, overlapping by factors $\frac {n}{k_{1} \cdot l_{1}}$ and $\frac {m}{k_{2} \cdot l_{2}}$, *l*_1_=*l*_2_=5. In the plots, each cell represents respective value (count, ratio, log *p*-value) in the window centered at the cell

This sample study (Fig. 2) shows significant over-representation of amyloidogenic sequences among peptides composed of larger hydrophobic amino acids. Non-amyloidogenic peptides are relatively more frequent among sequences made of smaller hydrophilic residues.

Many more examples are provided in the package documentation.

### Computational complexity

The essential time complexity of dataset processing with Quantiprot depends linearly on the number of sequences and on complexity of operations performed on each sequence. For example, amino acid conversions and basic operations such as averaging property value depends linearly on the sequence length, making the overall complexity in this typical use case dependent on the total size of the protein set. The most computationally demanding measures are RQA parameters which scale quadratically with the sequence length and linearly with the embedding level [14]. To limit computational burden, the specialized *RQAFeatureSet* object is recommended when calculating several RQA parameters in order to re-use results of previous calculations. Similarly, the specialized *NgramFeatureSet* object is provided for matching and counting all n-grams of a given length in a single pass through each sequence. When processing is performed with a sliding window, time complexity for each sequence depends on the sequence length and on complexity of operations performed in each window.

Practical estimates of time and memory required for simple tasks were obtained for a set of 20,188 sequences of the human reference proteome (average length 560, total size 11M amino acids) on a Debian Stretch-operated Fujitsu Celsius J550n desktop workstation equipped with Intel Xeon Env5 3.40GHz and 16GB Random-Access Memory (RAM). For example, conversion from amino acids to hydropathy indices took 1.5s on a single thread. While averaging over full sequence length did not increase the time, the same operation over the window of size 10 took 7.8s (the script consumed ca. 0.5GB RAM). In contrast, calculating the recurrence rate required almost 1h42m (and 0.9GB RAM) for full length sequences and just 10m for the window size of 10. Full bigram profiles for all sequences were calculated in 2m16s at the expense of 6GB RAM memory used. Depending on performed task and available memory of the user system, it may be advisable to process large sequence sets in a smaller chunks.

## Conclusions

Quantiprot is a powerful, flexible and extensible Python package for analyzing protein sequences in feature spaces defined by quantitative properties of amino acids and their tuples. The package provides a uniform interface to multiple methods in order to facilitate novel applications of quantitative analysis of protein sequences.

We propose three main fields of application of the Quantiprot package. First, quantitative characteristics can be used in alignment-free similarity searches, and in clustering of large and/or divergent sequence sets. Second, a feature space defined by quantitative properties can be used in comparative studies of protein families and organisms. Third, the feature space can be used for evaluating generative models, where large number of sequences generated by the model can be compared to actually observed sequences. For example, in a recent study the latter approach was used to investigate if an unequal crossing-over model assuming simple compositional pressure can explain observed recurrence patterns at highly variable sites of highly intrinsically conserved repeats in the NLR (Nucleotide-binding oligomerization domain (Nod)-like receptor) proteins in fungi [28].

Technically, whatever the application, the overture is to generate feature vectors whose elements numerically describe potentially relevant properties of sequences or sequence stretches. In the similarity search and clustering scenarios, the feature vectors are fed to a clustering or classification method, multitude of which are included in the *scikit-learn* package [29]. In the exploratory scenarios, it is sometimes practical to reduce dimensionality, e.g. using Principal Component Analysis, Linear Discriminative Analysis or their non-linear kernal versions, some of which are also available in the *scikit-learn*. Then the feature space can be analyzed for example using the Fisher’s exact test as proposed in the second example (see Results and discussion).

## Availability and requirements

**Project name:** Quantiprot

**Project home page:** https://git.e-science.pl/wdyrka/quantiprot

The repository provides the package, quick-start examples and command-line scripts for easy testing and performing essential processing. The package can also be installed from the Python Package Index by typing “pip install quantiprot”.

**Operating system(s):** any supporting Python 2.7 (tested on Linux)

**Programming language:** Python 2.7

**Other requirements:** matplotlib>=2.0.0, numpy>=1.11.0, powerlaw>=1.4.1, requests>=2.10.0, scipy>=0.17.0

**Licence:** The MIT License (https://opensource.org/licenses/MIT)

The datasets analysed during the current study are available in the Quantiprot repository, https://git.e-science.pl/wdyrka/quantiprot.

## Abbreviations

NLR: Nucleotide-binding oligomerization domain (Nod)-like receptor
RAM: Random-access memory
RQA: Recurrence quantification analysis
